# Mid-Term Clinical and Radiological Changes in the Ankle Joint in Varus Knee Osteoarthritis Following Total Knee Arthroplasty

**DOI:** 10.3390/jcm13164700

**Published:** 2024-08-10

**Authors:** Ali Said Nazlıgül, Metin Doğan, İsmail Duran, Joaquín Moya-Angeler, Mustafa Akkaya

**Affiliations:** 1 Department of Orthopedics and Traumatology, Sincan Training and Research Hospital, 06949 Ankara, Turkey; alisaid012@hotmail.com; 2 Department of Orthopedics and Traumatology, Ankara Bilkent City Hospital, 06800 Ankara, Turkey; drmetindogan@gmail.com (M.D.); ismailduran33@gmail.com (İ.D.); 3 Department of Orthopaedic Surgery and Traumatology, Hospital General Universitario Reina Sofía, 30003 Murcia, Spain; jmoyaangeler@gmail.com; 4 Instituto de Cirugía Avanzada de la Rodilla (ICAR), 30005 Murcia, Spain; 5 Department of Orthopedics and Traumatology, Güven Hospital, 06540 Ankara, Turkey

**Keywords:** arthroplasty, ankle joint, genu varum, knee osteoarthritis, total knee replacement

## Abstract

**Background/Objectives:** In patients with varus knee osteoarthritis, compensatory changes occur in the foot and ankle joints to compensate for the varus deformity of the knee. The aim of the study was to investigate the changes in the ankle of patients whose knee alignment was corrected with total knee arthroplasty (TKA) and to explore the clinical implications of these findings. **Methods**: In this retrospective observational study, we analyzed 204 knees of 179 patients who had regular follow-ups out of 431 patients who underwent TKA for varus knee osteoarthritis between January 2019 and July 2021. Patient demographics, body mass index, follow-up time, and radiographs were studied. The hip–knee–ankle (HKA) angle, joint line convergence angle (JLCA), talar tilt (TT) angle, ground talar dome (GT) angle, ground tibia plafond (GP) angle, and the American Orthopaedic Foot & Ankle Society (AOFAS) score were assessed preoperatively and at the last follow-up. **Results**: A total of 204 knees of 179 patients with a mean follow-up time of 32.50 ± 6.68 months were evaluated. It was found that the change in the HKA had a positive effect on the AOFAS score and a negative effect on the TT, GT, and GP angles. While the clinical score improved in 82 patients, it worsened in 8 patients. The age difference between the groups whose AOFAS score improved and worsened was statistically significant. **Conclusions**: The correction of the varus malalignment in the knee was shown to also improve the compensatory valgus in the foot and ankle over the mid-term, with a statistically significant improvement in the patients’ clinical ankle scores.

## 1. Introduction

Osteoarthritis (OA) is a chronic disease affecting a significant portion of the global population, characterized by the failed repair of joint damage due to stresses from abnormalities in joint or periarticular tissues [1]. Despite cartilage loss being a primary characteristic, OA affects the entire joint, resulting in the progressive degradation of the joint cartilage and the impairment of adjacent structures. Clinical manifestations include pain, stiffness, reduced joint mobility, and audible joint crepitus. In 2020, it was estimated that approximately 595 million people worldwide were afflicted with OA, a number expected to rise due to increasing life expectancy, obesity rates, and joint injuries [2]. This condition severely impacts the quality of life due to chronic pain and reduced mobility, leading to considerable morbidity [3]. Among the joints most affected by osteoarthritis, the knee ranks highest, followed by the hand and hip [4]. The knee, a crucial joint for daily activities, is subjected to substantial loads. As life expectancy increases and obesity rates rise, coupled with a growing number of joint injuries, the prevalence and incidence of knee osteoarthritis are expected to escalate [5,6]. The development and progression of knee OA are influenced by several risk factors, including age, gender, obesity, previous joint injuries, and occupational hazards involving repetitive joint loading [7]. Studies indicate that women are more susceptible to OA than men, and obesity is strongly associated with OA due to the increased mechanical stress on weight-bearing joints [8,9]. Additionally, participation in high-impact sports and activities can predispose individuals to osteoarthritis (OA) through cumulative joint trauma; however, there is no increased risk associated with recreational physical activities (e.g., running, swimming, or cycling) [10,11].

Biomechanical issues are major risk factors for knee osteoarthritis. Malalignment of the lower extremities disturbs knee joint biomechanics, with coronal plane malalignment accelerating the progression of knee OA [12]. Although valgus malalignment can occur, varus malalignment is more commonly associated with knee osteoarthritis [13]. The mechanical axis of the lower extremities extends from the hip to the foot. Advanced varus knee osteoarthritis is a long-term pathological process that alters the alignment of the entire lower extremity and can lead to degenerative changes in the ankle joint [14,15].

Total knee arthroplasty (TKA) is the gold standard treatment for end-stage knee OA globally. While patient satisfaction with TKA is generally high, dissatisfaction rates range from 10% to 25% [16]. Preoperative evaluations for TKA typically focus on knee alignment using plain radiographs, often overlooking adaptive and pathological changes in the hip and ankle joints. Varus or valgus malalignment of the knee can result in ankle malalignment and changes in the tibiotalar tilt angle [17]. Most TKA patients have some degree of flexibility in the ankle and hindfoot, which can result in changes after knee alignment adjustments.

Some studies suggest that varus deformity of the knee is compensated by the valgization of the ankle and subtalar joint [17,18]. However, other studies indicate that the alignment of the foot and ankle is not related to the alignment of the lower extremity [19]. There is a lack of research on the reversal of these changes following varus correction in the knee and their clinical impact on the ankle joint in the midterm. Understanding the changes in the ankle and hindfoot after correcting lower extremity varus deformities with TKA and examining their interrelationships and clinical outcomes is crucial. The aim of this study is to investigate the radiological changes in the coronal plane of the ankle after correcting varus malalignment with TKA and to explore the relationships between these changes and clinical outcomes. The findings are expected to highlight the changes in foot and ankle biomechanics in the treatment of varus knee osteoarthritis, emphasizing the factors that should be considered to achieve better clinical outcomes when planning treatment. Although there are studies in the literature on this subject, the research on mid-term results is limited, and there is still no consensus in this area.

## 2. Materials and Methods

**Figure 1:**
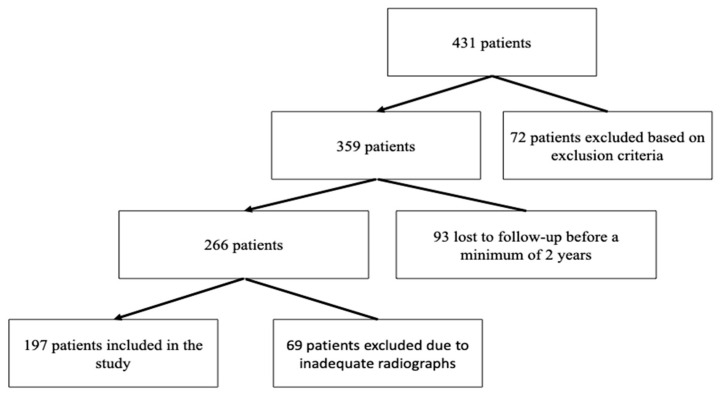
Flowchart of the patient enrollment process.

In this retrospective observational study, 204 knees of 179 patients out of 431 patients who underwent TKA for varus knee OA between January 2019 and July 2021 were included (Figure 1). Of the patients, 27 were men, and 152 were women. Of the 179 patients included in the study, 154 patients underwent TKA on one knee, and 25 patients underwent TKA on both knees. All patients underwent surgery using the same surgical technique, and the prosthetic design used for all patients was a posterior-stabilized fixed bearing. Routine preoperative and follow-up full leg standing anteroposterior and lateral knee radiographs were performed for each patient who underwent TKA. 

Patients were followed from 24 to 48 months, with an average follow-up of 32.50 ± 6.68 months. The study’s patient body mass index (BMI) measurements ranged from 21.8 to 46.1 kg/m^2^, with an average of 32.24 ± 4.23 kg/m^2^ (Table 1).

This study included patients aged 18 and older who were diagnosed with primary varus knee osteoarthritis and underwent primary posterior-stabilized TKA at our clinic between January 2019 and July 2021. The exclusion criteria were as follows: a history of inflammatory disease, secondary osteoarthritis diagnosis of the knee such as septic arthritis and post-traumatic arthritis, any surgical intervention history or requiring additional intervention on the same lower extremity included in the study, insufficient follow-up duration, and low-quality or incomplete preoperative and/or postoperative imaging.

This study was approved by the institutional ethics committee of Bilkent City Hospital (number: E1-22-3026) and was performed in accordance with the ethical standards of the 1964 Declaration of Helsinki and its subsequent amendments.

### 2.1. Radiological Evaluations

Preoperative and last visit full-leg standing radiographs were evaluated in each patient. Radiological measurements were performed using the picture archiving and communication system software. Two researchers independently measured the selected angles on all radiographs. The study utilized the average of the measured values. The hip–knee–ankle (HKA) angle was defined as the angle between a line drawn from the center of the femoral head to the femoral intercondylar notch and another line from the center of the tibial plateau to the mid-talar dome. The joint line convergence angle (JLCA) was measured as the angle between the femoral and tibial joint lines. The talar tilt (TT) angle was defined as the angle between the subchondral plate of the distal tibial articular surface and the talar dome, with the apex lateral angles positive and the apex medial angles negative. The ground talar dome (GT) angle was between the talar dome and a line parallel to the ground, and the ground plafond (GP) angle was between the subchondral plate of the distal tibial articular surface and a line parallel to the ground, both with apex lateral angles positive and apex medial angles negative.

**Figure 2:**
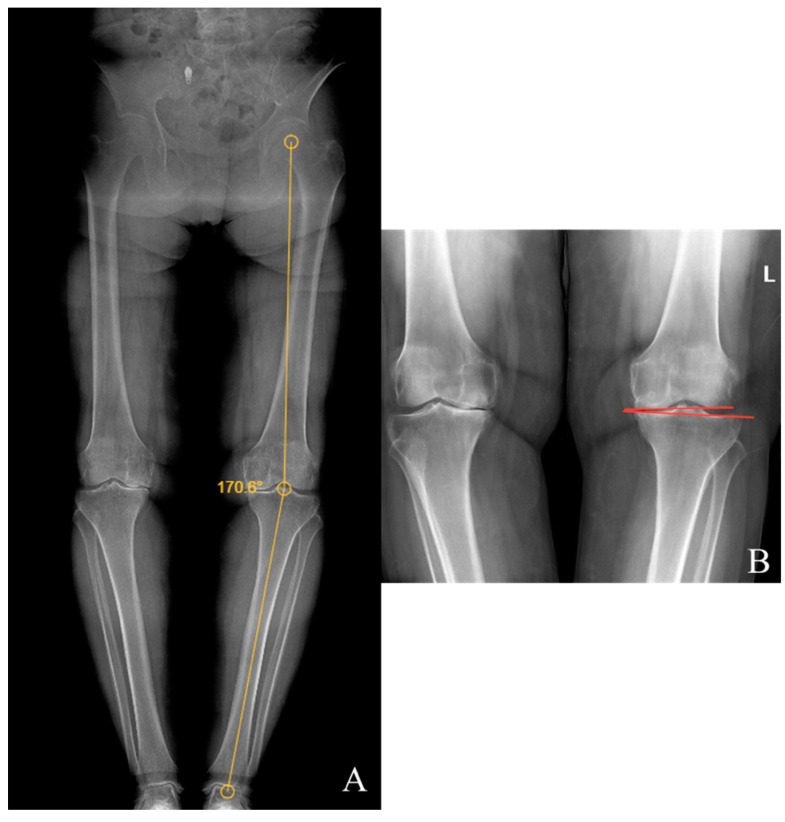
(**A**) Hip–knee–ankle angle; (**B**) knee joint line convergence angle.

**Figure 3:**
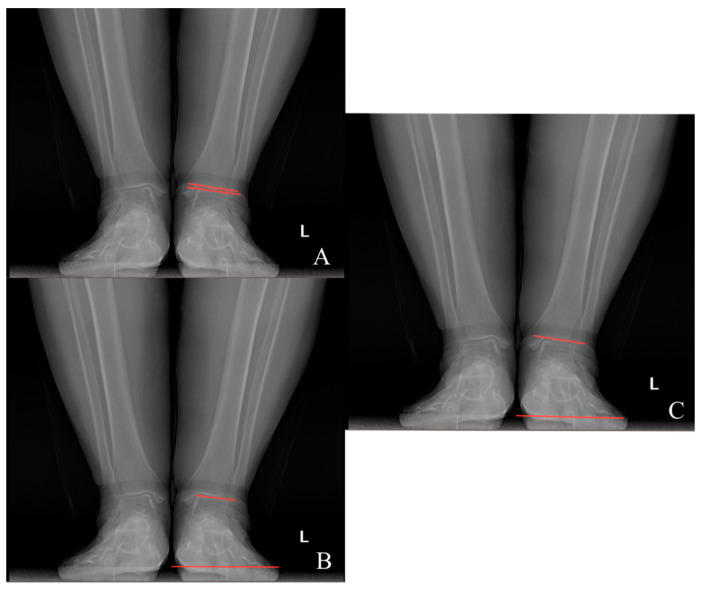
(**A**) Talar tilt angle; (**B**) ground talar dome angle; (**C**) ground tibia plafond angle.

The HKA, TT, JLCA, GT, and GP angles were measured on the patients’ radiographs (Figure 2 and Figure 3). The preoperative and last visit American Orthopaedic Foot & Ankle Society (AOFAS) scores were evaluated.

### 2.2. Statistical Analysis

The data were evaluated using IBM SPSS Statistics 26 (IBM Corporation, Armonk, NY, USA) at Bilkent City Hospital in February 2023. In the analysis of study data, frequency distribution (number, percentage) is reported for categorical variables, and descriptive statistics (mean, standard deviation) are reported for numerical variables. The independent samples t-test and chi-square test were used to determine whether there was a difference in measurements between groups in terms of improvement/deterioration, the dependent samples t-test to determine whether there was a difference between preoperative and last visit measurements, and Pearson correlation analysis to explore relationships between numerical measurements, revealing correlations between changes in the HKA angle and changes in the TT, GT, and GP angles. In addition, linear regression analysis was performed to determine the effect of changes in the HKA angle on other dependent variables, including the talar tilt (TT), ground talar dome (GT), ground tibia plafond (GP) angles, and AOFAS scores. *p* ≤ 0.05 was considered significant.

## 3. Results

Of the patients, 27 (15.1%) were male, and 152 (84.9%) were female. Of 179 patients, the right knee was operated in 70 (39.1%), the left knee in 84 (46.9%), and both knees in 25 (14%). The age of the patients was 65.57 ± 6.99 (47–83). The study’s patient body mass index (BMI) measurements ranged from 21.8 to 46.1 kg/m^2^, with an average of 32.24 ± 4.23 kg/m^2^ (Table 1).

Statistically significant differences were observed between the preoperative and last visit hip–knee–ankle angle (HKA), talar tilt angle (TT), knee joint line convergence angle (JLCA), ground talar dome angle (GT), ground tibia plafond angle (GP), and American Orthopaedic Foot & Ankle Society (AOFAS) score (Table 2, *p* < 0.05).

We observed a statistically significant positive correlation between the increase in the HKA angle and the change in the TT, GT, and GP angles and a negative correlation with the AOFAS score. (*p* ≤ 0.05). The results also demonstrated a statistically significant negative correlation between the AOFAS score and the TT, GT, and GP measurements (Table 3, *p* ≤ 0.05).

Changes in the HKA angle showed a statistically significant effect on the TT, GT, and GP angle changes and the AOFAS score change (*p* < 0.05). Accordingly, a 1 unit decrease in the HKA angle displayed a 0.052 unit decrease in the TT angle, a 0.420 unit decrease in the GT angle, a 0.483 unit decrease in the GP angle, and a 0.750 unit increase in the AOFAS score (Table 4).

While we did not observe a statistically significant difference between the AOFAS score improvement/deterioration groups in terms of gender and BMI (*p* > 0.05), our data showed a statistically significant difference in terms of age (*p* < 0.05). Accordingly, the age of patients whose AOFAS score worsened was higher than that of patients whose AOFAS score improved (Table 5).

Our results also showed no statistically significant difference between the AOFAS score improvement/deterioration groups in terms of the preoperative GT and GP measurements (*p* > 0.05), but a statistically significant difference in terms of HKA and TT measurements (*p* < 0.05) was observed. Accordingly, the mean preoperative TT was found to be negative in patients whose AOFAS score deteriorated (Table 6).

While we did not observe statistically significant differences between the last visit AOFAS score improvement/deterioration groups in terms of the GT angle change (*p* > 0.05), our data displayed a statistically significant difference in terms of the TT and GP angle change (*p* < 0.05). Accordingly, the change in the TT and GP angle is higher in those who improve than in those who worsen (Table 7).

## 4. Discussion

Our study provides valuable insight into the mid-term clinical and radiological changes in the ankle after TKA for varus knee OA. Correcting the varus deformities of the knee with TKA also addresses the compensatory ankle valgus. Our results demonstrate a significant correlation between the knee and ankle alignment, highlighting the interrelation of the lower extremity biomechanics.

Several studies have suggested that a varus malalignment of the knee is also associated with a compensatory valgus in the ankle. However, there is no consensus or sufficient research to determine whether correcting the knee’s varus deformity reverses this compensatory valgus in the ankle [17,18,20,21]. Norton et al. demonstrated an association between varus knee deformity and foot/ankle valgus and between valgus knee deformity and foot/ankle varus [22]. Similarly, Gao et al. showed that ankle alignment is associated with knee alignment both before and after surgery and that pre-operative malalignments in both the knee and ankle can be corrected simultaneously following TKA [20]. However, Chandler et al. found no correlation between preoperative lower extremity alignment and ankle alignment [19]. In our study, a significant relationship between varus knee osteoarthritis and ankle valgus alignment was demonstrated.

Many studies in the literature have reported an increase in AOFAS scores following a decrease in the HKA angle after TKA. Lee et al. observed a decrease in the GT, GP, and TT angles after TKA in patients with varus knee OA over a three-year follow-up period [23]. Palanisami et al. reported preoperative and final HKA angles of 18 ± 6.3 and 1.2 ± 1.9 degrees varus, respectively, with AOFAS scores improving from 59.2 ± 9.5 and 88.7 ± 5.9 [24]. Diao et al. found preoperative HKA angles of 9.15 ± 5.54 varus and final HKA angles 2.37 ± 3.05 varus, with AOFAS scores increasing from 76.50 ± 10.89 to 90.40 ± 6.05 [25]. However, the majority of studies in the literature have short-term follow-ups. The results of our study demonstrated that correcting knee varus led to an increase in the AOFAS scores at the mid-term follow-up. Furthermore, the correction of knee varus deformity also corrects compensatory valgus deformity in the ankle, resulting in both radiological and clinical improvements.

In the study by Lee et al., medial talar tilt was present in 39 of 128 patients, 18 of whom had ankle osteoarthritis [23]. However, the clinical scores of the patients were not available in this study. Wu et al. found no significant difference in the TT angle in relation to foot and ankle pain [26]. In our study, five out of eight patients with worsening AOFAS scores had medial talar tilt. In addition, a negative talar tilt angle was identified as a risk factor for worsening clinical scores (*p* = 0.007).

Wu et al. found no significant age difference but identified female gender and high BMI as risk factors for postoperative foot and ankle pain in TKA patients with varus knee OA [26]. In another study, Kim et al. observed that ankle pain after TKA was associated with residual varus deformity of the knee [27]. However, in our study, this association was not observed in patients with residual varus deformity of the knee. Our study found that older patients showed less improvement in clinical scores, which aligns with Diao et al.’s findings that compensatory mechanisms diminish with age. This suggests that age may be a limiting factor in the extent of improvement in ankle alignment and function after TKA [25]. This may explain why the older patients in our study showed less change in GP and TT angles and had worsened AOFAS scores.

There are studies in the literature showing changes in knee alignment and knee pain complaints after hip arthroplasty [28,29,30,31]. This demonstrates that all lower extremity alignments are interrelated. Kobayashi et al. suggest, that in patients presenting with knee and hip pain, addressing the hip first may result in a reduction in knee pain [31]. Similarly, our study shows that in patients with varus knee OA and ankle pain, the knee OA should be addressed first. However, in patients with negative talar tilt and those of advanced age, it is possible that the desired level of improvement may not be achieved or that the patient’s complaints may increase.

The main limitation of our study is the lack of a control group. Other limitations include the retrospective nature of the study, the unequal gender distribution, the narrow focus on coronal alignment, and the exclusive focus on varus deformity. The strengths of the study include the large patient cohort, long follow-up, and comprehensive clinical score evaluation. A consensus and sufficient studies on this topic are still lacking in the literature. It is therefore recommended that further research should prospectively include a larger patient population and a control group in order to provide more conclusive evidence.

## 5. Conclusions

The changes in the ankle after total knee arthroplasty in varus knee osteoarthritis have not been adequately clarified in the literature. In our study, the correction of the varus malalignment in the knee was shown to also improve the compensatory valgus in the foot and ankle over the mid-term, with a statistically significant improvement in patients’ clinical scores. The improvement in clinical scores in most patients after TKA in varus knee OA indicates that intervention for compensatory valgus is not necessary, and patients’ ankle pain may subside with the treatment of the varus knee OA.

While BMI and gender were not identified as risk factors for clinical score worsening in our study, age and a negative TT angle were found to be significant risk factors. However, the reasons for the deterioration in the clinical scores in some patients should be clearly identified, and appropriate plans should be made accordingly. The results we found need to be supported by prospective randomized controlled trials.

## Figures and Tables

**Table 1:** Demographic features.

		n	%
Sex	Male	27	15.1%
	Female	152	84.9%
Side	Right	70	39.1%
	Left	84	46.9%
	Both	25	14%
		mean	min–max
Age		65.57 ± 6.99	47–83
BMI		32.24 ± 4.23	21.8–46.1
Follow-up time (month)		32.50 ± 6.68	24–48

BMI: body mass index.

**Table 2:** Differences between preoperative and last visit measurements.

	Preoperative	Last Visit	t	*p*
HKA	13.32 ± 4.83	4.42 ± 2.97	28.475	0.000 *
JLCA	7.318 ± 2.23	0.30 ± 0.40	44.112	0.000 *
TT	0.76 ± 0.70	0.12 ± 0.60	20.758	0.000 *
GT	8.89 ± 4.71	4.28 ± 3.96	22.497	0.000 *
GP	9.61 ± 4.81	4.46 ± 3.89	23.266	0.000 *
AOFAS Score	93.62 ± 7.54	97.72 ± 5.21	−8.688	0.000 *

HKA: hip–knee–ankle angle, JLCA: joint line convergence angle, TT: talar tilt angle, GT: ground talar dome angle, GP: ground tibia plafond angle, AOFAS: American Orthopaedic Foot & Ankle Society. t: paired sample *t* test, *: *p* < 0.05.

**Table 3:** Relationship between measurements.

		HKA Angle	TT Angle	Ground Talus Angle	Ground Plafond Angle	AOFAS
HKA	r	1	0.052	0.641	0.682	−0.498
	*p*		0.000 *	0.000 *	0.000 *	0.000 *
TT	r		1	0.397	0.486	−0.197
	*p*			0.000 *	0.000 *	0.005 *
GT	r			1	0.950	−0.233
	*p*				0.000 *	0.001 *
GP	r				1	−0.287
	*p*					0.000 *
AOFAS Score	r					1
	*p*					

HKA: hip–knee–ankle angle, TT: talar tilt angle, GT: ground talar dome angle, GP: ground tibia plafond angle, AOFAS: American Orthopaedic Foot & Ankle Society. r: Pearson correlation coefficient, *: *p* < 0.05.

**Table 4:** Effects of HKA changes on measurements.

	Unstandardized Coefficient		Standardized Coefficient	t	*p*	95.0% CI	
	B	Standard Error	Beta			Lower Limit	Upper Limit
**TT**							
(Constant)	0.176	0.059		3.005	0.003	0.061	0.292
HKA	0.052	0.006	0.528	8.843	0.000	0.064	0.040
(F: 78.190, *p*: 0.000 *, R^2^: 0.279)							
**GT**							
(Constant)	0.867	0.352		2.462	0.015	0.173	1.562
HKA	0.420	0.035	0.641	11.883	0.000	0.490	0.351
(F: 141.214, *p*: 0.000 *, R^2^: 0.411)							
**GP**							
(Constant)	0.850	0.362		2.344	0.020	0.135	1.564
HKA	0.483	0.036	0.682	13.261	0.000	0.555	0.411
(F: 175.842, *p*: 0.000 *, R^2^: 0.465)							
**AOFAS Score**							
(Constant)	2.586	0.915		2.825	0.005	0.781	4.390
HKA	−0.750	0.092	−0.498	−8.159	0.000	−0.569	−0.932
(F: 66.566, *p*: 0.000 *, R^2^: 0.248)							

HKA: hip–knee–ankle angle, TT: talar tilt angle, GT: ground talar dome angle, GP: ground tibia plafond angle, AOFAS: American Orthopaedic Foot & Ankle Society. B: regression coefficient *: *p* < 0.05.

**Table 5:** AOFAS score improvement/deterioration groups’ demographic differences.

		Improvement (n = 82)		Deterioration (n = 8)		Test	*p*
		n	%	n	%		
Sex	Male	11	13.4	0	0.0	1.223 ^x^	0.589
	Female	71	86.6	8	100.0		
Age (mean ± sd)		64.57 ± 6.57		69.88 ± 3.80		−2.240 ^t^	0.028 *
BMI (mean ± sd)		33.47 ± 4.66		32.31 ± 1.36		1.639 ^t^	0.112

AOFAS: American Orthopaedic Foot & Ankle Society, BMI: body mass index. ^x^: chi-square test, ^t^: independent sample *t* test, *: *p* < 0.05.

**Table 6:** Preoperative measurements of AOFAS Score improvement/deterioration groups.

	Improvement (Preoperative)	Deterioration (Preoperative)	t	*p*
HKA	16.22 ± 4.50	12.02 ± 4.63	2.510	0.014 *
TT	0.99 ± 0.55	−0.58 ± 1.17	3.732	0.007 *
GT	9.93 ± 4.46	7.58 ± 9.01	0.730	0.488
GP	10.90 ± 4.63	7.00 ± 7.91	1.372	0.210

HKA: hip–knee–ankle angle, TT: talar tilt angle, GT: ground talar dome angle, GP: ground tibia plafond angle, AOFAS: American Orthopaedic Foot & Ankle Society. t: independent sample *t* test, *: *p* < 0.05.

**Table 7:** Change in measurements between the last visit AOFAS score improvement/deterioration groups.

		Mean	sd	t	*p*
TT	Improvement	0.729	0.407	2.687	0.009 *
	Deterioration	0.325	0.392		
GT	Improvement	5.413	3.094	0.487	0.627
	Deterioration	4.850	3.441		
GP	Improvement	6.111	3.244	2.217	0.029 *
	Deterioration	3.400	3.902		

TT: talar tilt angle, GT: ground talar dome angle, GP: ground tibia plafond angle, AOFAS: American Orthopaedic Foot & Ankle Society. t: independent sample *t* test, *: *p* < 0.05.

## Data Availability

The data presented in this study are available on request from the corresponding author.
